# Less invasive treatment option for renal carcinoma with venous tumor thrombus

**DOI:** 10.3325/cmj.2014.55.265

**Published:** 2014-06

**Authors:** Zoltán Nagy, József Pánovics, Attila Szendrői, Attila Marcell Szász, László Harsányi, Imre Romics

**Affiliations:** 1 1st^.^ Department of Surgery, Semmelweis University, Budapest, Hungary; 2 Department of Urology, Semmelweis University, Budapest, Hungary; 3 2nd Institute of Pathology, Semmelweis University, Budapest, Hungary

## Abstract

**Aim:**

To retrospectively analyze patients treated by renal tumor and venous tumor thrombus (VTT) removal and to introduce a less stressful and safer surgical method without thoracotomy in Neves level 3 cases.

**Methods:**

From 2002 to 2011, 33 patients underwent surgery for renal cell cancer combined with tumor thrombus of the inferior vena cava. Preoperative symptoms, tumor-node-metastasis classification of tumors, thrombus extension classified by Neves and Zincke system, types of surgical interventions, complications, postoperative management, and survival results were analyzed.

**Results:**

Ten patients had level 1, 17 had level 2, and 6 had level 3 thrombi according to Neves and Zincke. In 5 patients with level 3 thrombi, the liver was mobilized without thoracotomy and in 1 patient endoluminal occlusion was utilized. There was no intraoperative mortality. The median survival time of 10 patients who died during follow-up period was 36.6 months (range, 1-116 months).

**Conclusion:**

Renal cell cancer complicated with tumor thrombus without metastasis can be curable by performing a complete resection. The thrombus level determines the surgical approach and method. Our results confirm that level 3 caval vein tumor thrombus can be safely surgically treated by laparotomy with liver mobilization. Thoracotomy, use of cardiopulmonary bypass, and hypothermic circulatory arrest can be avoided with adequate liver- and vascular surgery methods.

In 4%-15% of renal cell cancer cases, tumor thrombus is formed in the renal vein (RV) and later in the inferior vena cava (IVC), and in 1% the thrombus spreads into the right atrium (1-5). For advanced stage renal cell cancer, a radical nephrectomy with removal of the tumor thrombus is required (1,2,4,5). Over the past 40 years, the level 3 and 4 thrombi according to Neves and Zincke system have been surgically treated through thoracoabdominal incision with extracorporal circulation with or without hipothermia (3,5). Nowadays, in order to reduce the incision size, minimally-invasive techniques are used. In selected cases, laparoscopic surgery may also be performed (6,7). The thrombus extension classified by Neves and Zincke system is the following: level 1 (renal), level 2 (infrahepatic inferior vena cava), level 3 (retrohepatic inferior vena cava), and level 4 (suprahepatic, supradiaphragmatic inferior vena cava, and atrial) (8) (Figure 1). Our aim was to introduce a less aggressive surgical approach without thoracotomy and evaluate its efficacy in high risk patient population with level 3 venous tumor thrombus (VTT).

**Figure 1:**
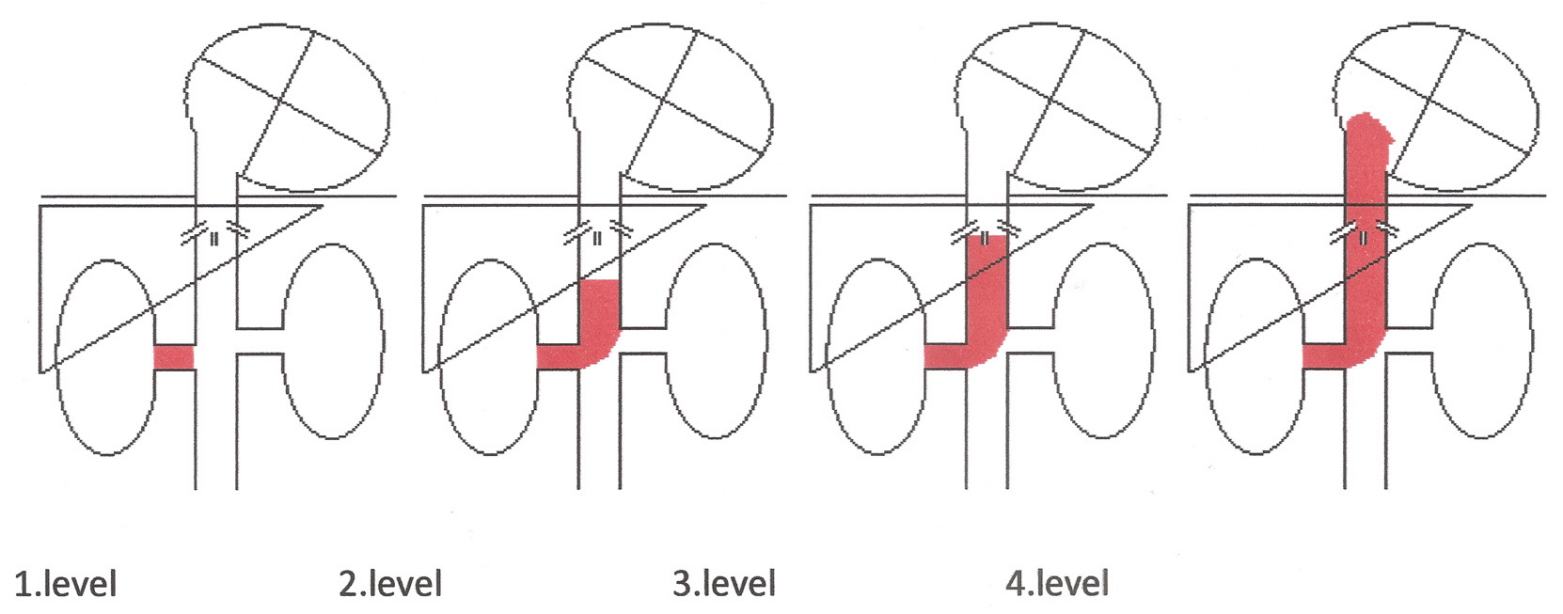
The limit of venous tumor thrombus extension, classified according to the Neves and Zincke system (8).

## Material and methods

### Patient population

Between 2002 and 2011, at the Department of Urology, Semmelweis University 968 surgeries of renal cell cancer were performed. We studied the 33 cases in which renal cell cancer was combined with tumor thrombus of the RV and the ICV. Among them, there were 12 women (36.4%) and 21 men (63.6%), with the average age of 60.5 years (31-79 years, standard deviation 9.138).

### Preopeartive diagnostics

Before each surgery, abdominal ultrasound and CT scan were performed, and in 21 cases MRI was performed (9-11). 

In level 2 cases, surgical procedure included the transperitoneal surgery through Chevron (subcostal)-incision: exploration, ligation of the renal artery, exclusion of the section above the IVC thrombus and the section below the renal veins followed by the exclusion of the intact renal vein, longitudinal cavotomy or the excision of the orifice of renal vein on the affected side, thrombectomy, flushing of the caval vein, de-gassing, lateral clamping of the cavotomy with Satinsky forceps, release of the exclusion, cavotomy closure with running suture, and nephrectomy. In level 1 cases, the cava was not involved, therefore the surgical intervention was less complicated, however in the level 3 cases the mobilization of the liver was required (11-13). The histological rating of the tumor was carried out according to the classification of Heidelberg, the staging was performed based on the 2010 tumor-node-metastasis (TNM) classification, and the histological grade was characterized according to Fuhrman (14,15).

## Results

Among 33 patients, there were 10 patients with level 1, 17 with level 2, and 6 with level 3. In these patients, Neves classification, number of cases, surgery type, and surgical time, the blood loss during surgery, intraoperative complications, reoperation and perioperative death was analyzed (Table 1)

**Table 1:** Summary of the surgical data and complications

	Neves classification level		
	1	2	3
Number of cases	10	17	6
Surgery type	Exclusion in 1 case	Exclusion in 13 cases	5 cases of exclusion after liver mobilization, 1 endoluminal occlusion
Surgical time (minutes)	203	220	215
Surgical blood loss (mL)	760	1350	1570
Intraoperative complications	1 pulmonary embolism, 1 splenic injury	1 ureter-injury, 2 cases of pulmonary embolism, 1 case of bleeding from the kidney’s bed, 1 acute heart failure	0
Reoperation	0	2	0
Perioperative death	0	1 postoperative	0

During the course of surgeries, preoperative renal artery occlusion was performed in 5 cases with interventional radiological methods (selective embolization). The clamping of the caval vein was not necessary in 13 cases. Among them, there were 9 patients with level 1 VTT. In these cases, the thrombus reached only the caval vein. In 4 patients, level 2 VTT was pushed back from the cava to the renal vein. The clamping of the IVC was performed in 20 patients, with an average clamping time of 8.4 minutes (3-20 minutes). The mobilization of the liver was performed in 5 cases, all of them with level 3 VTT. After mobilization, the IVC was made visible, it was well palpable around its circumference in all cases, and therefore it could be safely clamped.

We surgically treated 6 patients with level 3 VTT. In 4 cases, the clamping was made bellow the hepatic veins. In these cases, there was no bleeding from the liver to the cavotomy. In 1 case, the tumor thrombus reached a higher position than in previous 4 cases, therefore the clamping was performed above the liver and the retrograde bleeding was reduced with the Pringle-maneuver. In 1 case, thrombectomy was performed using a Foley-catheter to overcome the endoluminal occlusion. The catheter easily passed by the solid tumor thrombus, there was no embolization, and the operative time was shorter than in other cases. The cavotomy, as well as the excision of the orifice of the affected renal vein, was closed with running suture using a Satinsky forceps for lateral clamping. There was no postoperative cava occlusion. Lymph node block dissection was performed in only 5 cases, in case of palpable enlarged lymph nodes. The infiltration of the caval vein wall was not observed in any of the cases, therefore cava resection was not needed. The average operative time was 3 hours and 34 minutes (2 hours-5 hours and 45 minutes). The median intraoperative blood loss was 1075 mL (200-3500 mL), which was substituted with 3.4 U (0-12 U) of red blood cell mass transfusion.

Three reoperations were performed, one due to an injury of the contralateral ureter, one because of the bleeding from the removed kidney’s bed, and one because of splenic injury. At the beginning of the less invasive surgical procedure, in 2 cases the clamping of vena cava was not performed, leading to pulmonary embolism. One occurred in the course of operation and the other at the time of the extubation. These patients received anticoagulant therapy and fully recovered.

A patient, who suffered from multiple vascular disease, died on the second postoperative day. The cause of death was necrosis of the small bowel induced by the occlusion of the superior mesenteric artery. Other postoperative complications were not detected (Table 1)

Patients spent a median of 14.3 days (3-35 days) in hospital. Based on the Heidelberg classification, in 30 cases histological examination confirmed clear cell RCC and 1 case of sarcomatoid carcinoma, 1 case of anaplastic carcinoma, and 1 case of primitive neuroectodermal carcinoma. The tumor was found 22 times on the right side and 11 times on the left side. Thirteen tumors were found in the upper third, 13 in the middle third, and 7 in the lower third of the kidney.

At the time of the operation, 11 patients (33.3%) had distant metastases, mostly in the lungs and the retroperitoneum. Other sites included the liver and the mediastinum. The maximum diameter of the renal tumor was on average 101 mm (50-280 mm). According to the TNM classification, 31 tumors were T3 and 2 cases were T4. Based on the Fuhrman staging there was 1 G1, 11 G2, 13 G3, and 8 G4-tumors.

The median tumor thrombus length was 54 mm (10-130 mm). There was no intraoperative mortality. One patient died postoperatively (3%). The patients were monitored every 6 months after surgery. Serum creatinine, urea, and electrolyte levels were determined and abdominal ultrasound, chest, and abdominal CT examinations carried out. Survival time was determined in accordance with the date of death or the last follow-up date. The median follow-up period was 30 months (1-116 months).

For RCC, the patients were treated with subcutaneous Interferon-1α 9 million units 3 times a week combined with 0.1 mg/kg body weight of intravenous vinblastine once a month. The duration of this therapy was determined by the general condition of the patients and the outcome of the disease – ideally it lasted for 1 year. In one case, due to the poor general condition of the patient, the postoperative oncologic treatment was disregarded. After 2008, 5 patients with distant metastases were treated with tyrosine kinase inhibitors.

**Figure 2:**
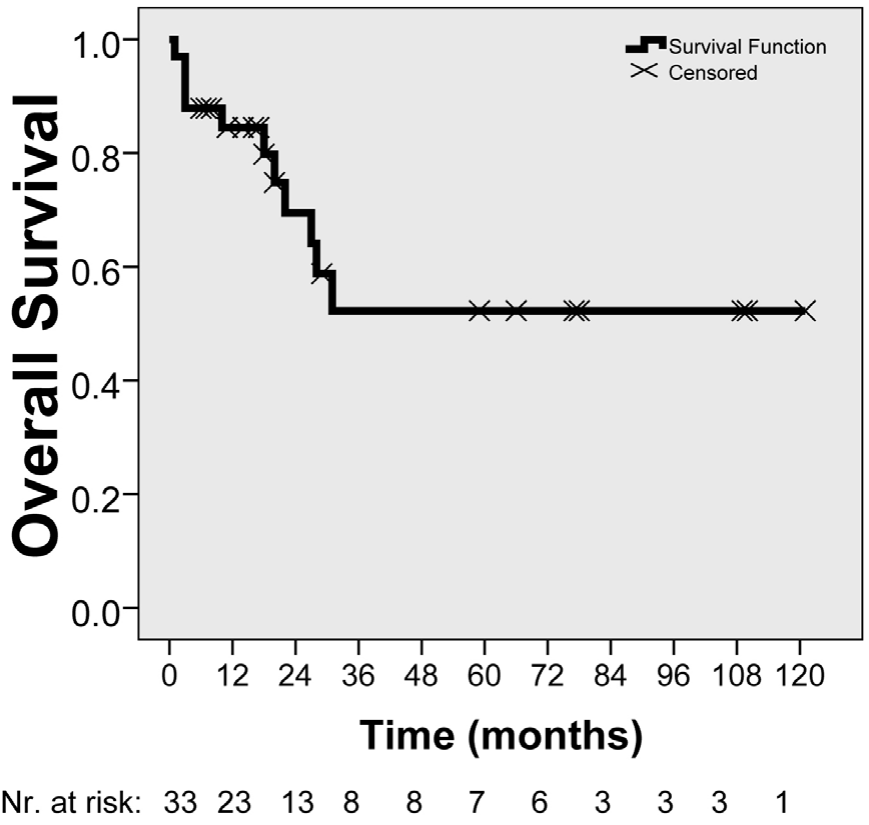
The survival rates calculated by Kaplan-Meier survival estimates for 33 patients.

Seven of the 11 patients with distant metastases (33.3%) undergoing surgery died in an average of 12.1 months (3-19 months). Of the patients with no metastases at the time of surgery, 3 died, with median survival of 26.7 months (22-31 months). All deaths were caused by the postoperative progression of the underlying disease. Twenty-two patients (66.6%) were alive at the end of the follow-up. Four of them developed metastases following the surgery in an average time of 14.5 months (9-24 months). Eighteen patients without metastases had median survival time of 41.5 months (1-116 months) following surgery. The median survival rate for 33 patients calculated by the Kaplan-Meier survival was 18 months (range, 0-121) (Figure 2).

## Discussion

The less invasive surgical approach used in this study reduced the complication rate, surgical time, and blood loss. In 5 cases, we preoperatively embolized the renal artery to reduce the tumor and the VTT size and to collapse the collateral veins (16-19). 

We did not notice complications like systemic reaction, embolization of another organ, and embolization of the tumor by disintegrating VTT reported by other surgical teams (20,21). 

The surgical plan depends on the VTT level. Previously, the tumor thrombus levels 3 and 4 were treated using right thoracolaparotomy. Nowadays thoracolaparotomy is less frequent and the combination of median sternotomy and laparotomy is increasingly used instead. The Chevron-incision provides opportunity for liver mobilization, therefore the VC clamping can be done without thoracotomy. While initially the Chevron-incision was used only for the thrombi localized below the diaphragm, now it is also used for those localized above the diaphragm. As a result, moderate forms of tumor thrombus level 4 can be treated by laparotomy (12,13).

The introduction of cardiopulmonary bypass (CPB) and hypothermic circulatory arrest (HCA) has allowed performing the dissection in a virtually blood-free area (22,23). The use of these methods has reduced the risk of embolization, but it has also increased blood-loss, operative time, ischemic damage of the brain and other organs, and the incidence of postoperative coagulopathies. In order to avoid complications of CPB, the depth of hypothermia and the antegrade heart and brain perfusion are reduced, and the intermittent supraceliac aorta exclusion is performed (24,25). In case of a thrombus only minimally extending above the diaphragm, veno-venous bypass was introduced. The perioperative complication rate using veno-venous bypass was 17%, and with the use of CPB + HCA the rate was 31% (3). Due to the development of surgical techniques, some work teams use extracorporal circulation only in the case of large atrium thrombi or if the thrombus is attached to the atrial wall (24,25).

The limitation of the study is that at the beginning of our practice we had to perform preoperative selective embolization in the cases when it was needed. 

Our results show that level 3 caval vein tumor thrombus can be removed by a less aggressive surgical approach, underlining the benefits of a surgical intervention without thoracotomy.
